# Solvent-Dependent Electrical Characteristics and Mechanical Stability of Flexible Organic Ferroelectric Field-Effect Transistors

**DOI:** 10.3390/mi10110727

**Published:** 2019-10-28

**Authors:** Do-Kyung Kim, Hyeonju Lee, Xue Zhang, Jin-Hyuk Bae, Jaehoon Park

**Affiliations:** 1 School of Electronics Engineering, Kyungpook National University, Daegu 41566, Korea; kdk7362@naver.com; 2 Department of Electronic Engineering, Hallym University, Chuncheon 24252, Korea; zoozs123@naver.com; 3 College of Ocean Science and Engineering, Shandong University of Science and Technology, Qingdao 266590, China

**Keywords:** flexible electronics, ferroelectric field-effect transistor, mechanical stability, solvent

## Abstract

Flexible organic ferroelectric field-effect transistors (Fe-FETs) have attracted attention for next-generation memory applications. A fundamental understanding of the electrical properties and mechanical stability of transistors is a prerequisite to realizing practical flexible electronics. Here, we demonstrate the solvent-dependent electrical characteristics and mechanical stability of flexible Fe-FETs. Poly(vinylidene fluoride-trifluoro-ethylene) (P(VDF-TrFE)) based Fe-FETs were fabricated by using dimethylformamide (DMF) and methyl ethyl ketone (MEK) solvents on a polyimide substrate. P(VDF-TrFE) from DMF formed a smoother surface than a surface from MEK; the surface property greatly affected the electrical properties and mechanical stability of the devices. Larger hysteresis and higher mobility were obtained from Fe-FET using DMF compared to those characteristics from using MEK. Furthermore, Fe-FET using DMF showed lower degradation of on-current and mobility under repetitive mechanical stress than an MEK-based Fe-FET, due to its excellent semiconductor-insulator interface. These results will guide appropriate solvent selection and contribute to the improvement of flexible Fe-FET electrical properties and mechanical stability in the next generation of memory devices.

## 1. Introduction

Nonvolatile memory functions that can be programmed, read out, and erased electrically are required in almost all electronic devices [1]. In this regard, ferroelectric field-effect transistors (Fe-FETs) have received significant attention because of their outstanding functionalities in a small size, including nonvolatile data retention, rewritability, non-destructive read-out, and fast programming [2,3,4]. Even though inorganic Fe-FETs have been developed and have achieved suitable memory performance for practical use, several important issues, such as the nature of inorganic materials and fabrication temperatures, still remain as obstacles to the implementation of advanced devices [5]. Thus, interest in organic ferroelectric materials based Fe-FETs has emerged recently due to their low fabrication temperatures and flexible, stretchable organic materials [6,7,8]. Poly(vinylidene fluoride-trifluoro-ethylene) (P(VDF-TrFE)), random copolymers of poly(vinylidenefluoride) with trifluoroethylene, is one of the most promising and general organic ferroelectric materials for the gate dielectric in Fe-FETs, owing to its relatively low processing temperature, solubility, and spontaneous polarization effect [5,9,10,11]. However, organic Fe-FETs fabricated on a flexible substrate still incur critical problems in electrical performance, even though useful performance from these Fe-FETs has been achieved when fabricated on a rigid substrate like glass or silicon. Generally, an unstable fabrication process, a low glass transition temperature, and a low water vapor transition rate caused by plastic substrates may lead to poor electrical properties in transistors, and repetitive mechanical stresses during flexible device use may cause unstable device operation by generating physical and chemical defects in thin-films and transistors [12,13,14,15]. Therefore, a deep understanding of the electrical characteristics and mechanical stability of flexible Fe-FETs are required for their utilization in flexible electronic applications. Among many factors that affect the electrical properties and stability of thin-films and devices, solvent is of prime importance, particularly in solution-processed transistors. Previously, Knotts et al. reported the significance of dipole moments of solvents on ferroelectric properties of P(VDF-TrFE) films in Fe-FETs; higher dipolar solvents result in higher remnant polarizations in ferroelectric insulator films [16]. Nevertheless, it is still required to clarify how the boiling point of solvents for ferroelectric insulators affects the performance of Fe-FETs. This is important because boiling point and chemical properties of solvents inevitably dictate the morphological properties, such as grain size and surface roughness, of solution-processed ferroelectric films [17,18,19,20,21]. In particular, the effects of solvents on the electrical characteristics and mechanical stability of flexible organic Fe-FETs should be considered.

In this study, we investigate the solvent-dependent electrical characteristics and mechanical stability of Fe-FETs by fabricating P(VDF-TrFE) based flexible Fe-FETs using dimethylformamide (DMF) and methyl ethyl ketone (MEK) solvents on a polyimide (PI) substrate. The P(VDF-TrFE) from MEK solvent exhibits a rougher surface compared with the P(VDF-TrFE) from DMF solvent. The surface roughness of a ferroelectric insulator critically affects the electrical properties and mechanical stability of flexible Fe-FETs. P(VDF-TrFE) based flexible Fe-FETs using DMF solvent show better memory and electrical performance than Fe-FETs from MEK; the improved properties include larger hysteresis, higher drain current, and higher field-effect mobility. Furthermore, Fe-FETs using MEK solvent exhibit inferior mechanical behavior, owing to their surface roughness induced semiconductor-insulator interface instability, while Fe-FETs using DMF solvent exhibit stable electrical properties after 100 cycles of bending stress by virtue of their smooth insulator surface.

## 2. Materials and Methods

**Figure 1:**
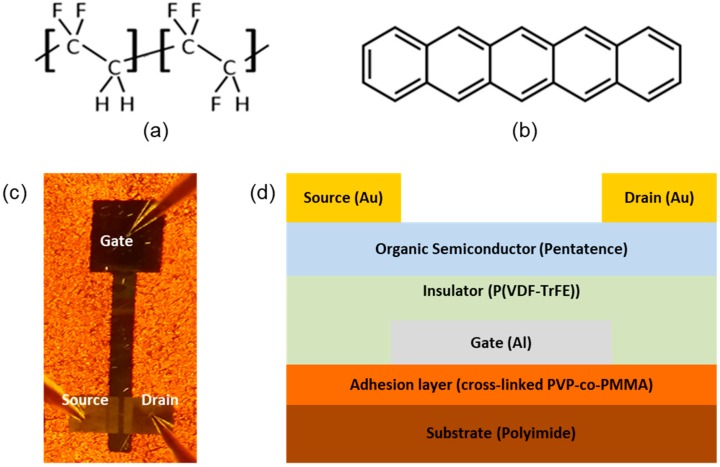
Molecular structures of (**a**) P(VDF-TrFE) and (**b**) pentacene; (**c**) optical microscopy image of a measured flexible Fe-FET; (**d**) cross-section schematic of flexible Fe-FET.

For this study, bottom-gate and top-contact structured pentacene Fe-FETs were fabricated on PI substrates. Prior to Fe-FET fabrication, the PI substrates were cleaned with acetone, 2-propanol, and deionized water in sequence for 10 min each. In our experiments, a polymeric adhesion layer was formed on the PI substrate so that the Al gate electrode and the P(VDF-TrFE) ferroelectric layer could be better adsorbed onto the PI substrate. As a polymeric adhesion material, poly(4-vinylphenol-co-methyl methacrylate) (PVP-co-PMMA) was dissolved in propylene glycol methyl ether acetate (PGMEA) with a cross-linking agent of poly(melamine-co-formaldehyde) (PMF). The concentration ratios of PVP-co-PMMA and PMF in the PGMEA solvent were each 3 wt %. To form the adhesion layer, the PVP-co-PMMA solution was spin-coated at 2000 rpm for 35 s onto the cleaned PI substrates, and then baked on a hot plate at 210 °C for 1 h to induce cross-linking between the PVP-co-PMMA chains. For the gate electrode, a 50-nm-thick Al layer was thermally deposited at a rate of 0.1 nm/s onto the adhesion layer through a shadow mask. Here, the individual 10 wt % P(VDF-TrFE) solutions were prepared in DMF and MEK, respectively; the molecular structure of P(VDF-TrFE) is shown in Figure 1a. For the gate insulators, the P(VDF-TrFE) solution from DMF or MEK solvents were spin-coated at 2000 rpm for 30 s onto the substrates, and then baked on a hot plate at 140 °C for 2 h. To form an organic semiconductor layer, a 50-nm-thick pentacene film was thermally deposited onto the prepared insulators at a deposition rate of 0.1 nm/s; the molecular structure of pentacene is shown in Figure 1b. Finally, 50-nm-thick Au source and drain electrodes were thermally deposited onto the pentacene films through a shadow mask at a deposition rate of 0.1 nm/s. The channel length and width were 100 and 800 μm, respectively. A photograph and a schematic cross-sectional view of our device are shown in Figure 1c,d, respectively.

## 3. Results and Discussion

**Figure 2:**
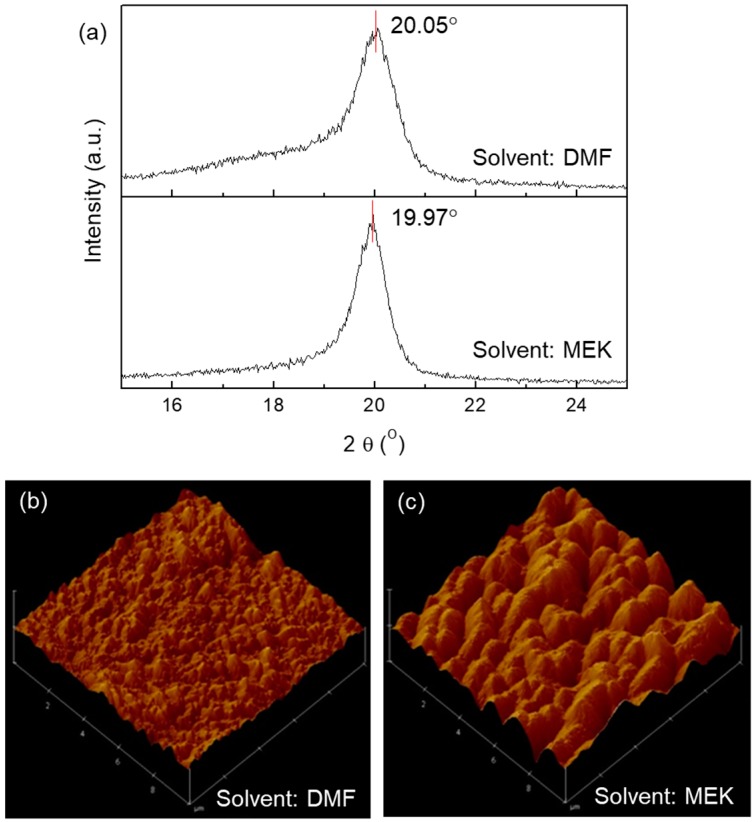
(**a**) XRD patterns of P(VDF-TrFE) films from DMF (top) and MEK (bottom) solvents; AFM images of P(VDF-TrFE) surfaces from (**b**) DMF and (**c**) MEK solvents.

The effects of solvent on the molecular and morphological properties of P(VDF-TrFE) insulator were investigated by using X-ray diffraction (XRD) and an atomic force microscope (AMF). Figure 2a shows XRD patterns in the out-of-plane mode of P(VDF-TrFE) films from DMF (top) and MEK (bottom) solvents. In P(VDF-TrFE) films from DMF and MEK solvents, XRD peaks were observed at 20.05° and 19.97°, respectively, corresponding to the typical range of the β-phase. This indicates that both films have suitable dielectric and ferroelectric properties for the insulation of Fe-FETs by virtue of a switchable dipole moment from β-phase crystal structure [22,23]. Boiling point is one of the most influential factors that affect morphology, crystallinity, and electrical characteristics of organic semiconductors [24,25]. The P(VDF-TrFE) from MEK solvent, which has a low boiling point of 79 °C, had a sharp XRD peak and a small full width at the half maximum (FWHM) of the XRD pattern; this was small compared to the P(VDF-TrFE) from DMF solvent, which has a higher boiling of 152 °C. This result indicates that a low boiling point solvent promotes grain formation or crystallization of P(VDF-TrFE) film during the annealing process for activation, resulting in large crystalline domains [26]. This is corroborated by morphological analysis of P(VDF-TrFE) films from DMF and MEK solvents. The AFM images of P(VDF-TrFE) films from DMF and MEK solvents are shown in Figure 2b,c, respectively. The root-mean-square (RMS) roughness of P(VDF-TrFE) films from DMF and MEK solvent were 14.95 nm and 23.24 nm, respectively. Large grains were observed in P(VDF-TrFE) film from MEK solvent compared to the grain from DMF solvent. Large grains of P(VDF-TrFE) film from MEK solvent induce high surface roughness which hinders the charge transport of carriers at the interface of insulator and semiconductor. A relatively smooth surface was formed on the P(VDF-TrFE) film from DMF solvent.

**Figure 3:**
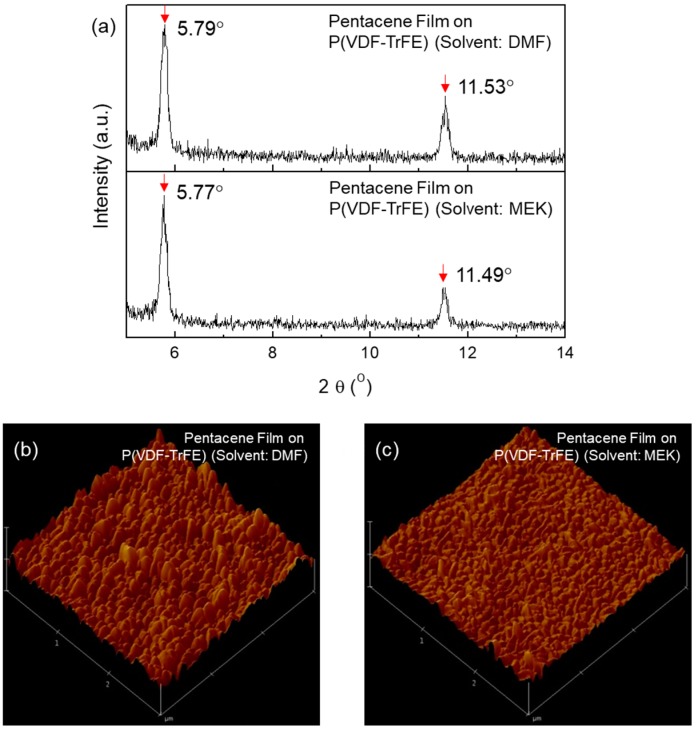
(**a**) XRD patterns of pentacene films on P(VDF-TrFE) from DMF (top) and MEK (bottom) solvents; AMF images of pentacene films on P(VDF-TrFE) from (**b**) DMF and (**c**) MEK solvents.

Figure 3a shows XRD patterns in the out-of-plane mode of pentacene films on P(VDF-TrFE) films from DMF (top) and MEK (bottom) solvent. Distinct (001) and (002) diffraction peaks were observed in both cases. The FWHM of the (001) diffraction peak is also equal to 0.16° in both cases. The crystallinities of the pentacene films on P(VDF-TrFE) from DMF and MEK did not show any significant difference. Figure 3b,c display the AFM images of pentacene films on P(VDF-TrFE) films from DMF and MEK solvents, respectively. Due to the poor morphology of the P(VDF-TrFE) film from MEK, the grains of pentacene grown on the P(VDF-TrFE) film from MEK were relatively small compared to those grown on the P(VDF-TrFE) film from DMF. The RMS roughness of pentacene films on P(VDF-TrFE) films from DMF and MEK solvents were approximately 20.97 nm and 24.01 nm, respectively.

**Figure 4:**
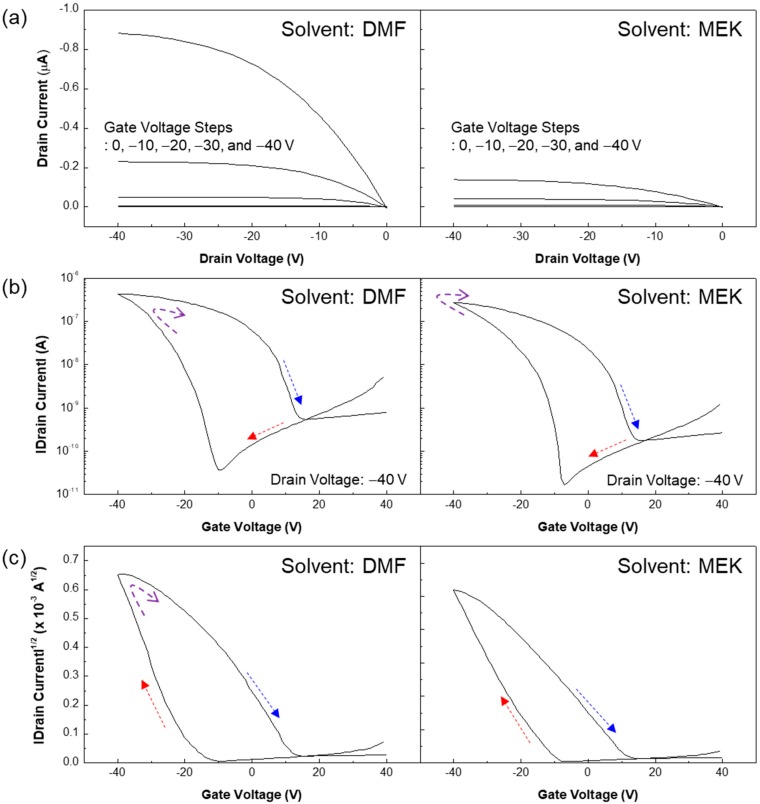
(**a**) Output characteristic curves of flexible Fe-FETs using DMF (left) and MEK (right) solvents; (**b**,**c**) Transfer characteristic curves (SQRT |drain current| and log |drain current| versus gate voltage, respectively) of flexible Fe-FETs using DMF (left) and MEK (right) solvents.

Figure 4a shows the output characteristics of flexible Fe-FETs using DMF (left) and MEK (right) solvents. A higher drain current was measured in the Fe-FET using DMF solvent than measured in the Fe-FET using MEK solvent. Here, the high drain current of the Fe-FET using DMF solvent is mainly attributed to the smoother surface morphology of the insulator and therefrom larger pentacene grains, whereas the effect of P(VDF-TrFE) grain size on the electrical characteristics of Fe-FETs seems less dominant [27,28]. The morphology of the insulator is one of the most important properties for charge transport. Figure 4b,c show the transfer characteristics of flexible Fe-FETs from DMF (left) and MEK (right) solvents. The hysteresis which defines the binary 1 and 0 states was obtained by supplying a sweeping gate voltage for all Fe-FETs, as shown in Figure 4b. A hysteresis loop was observed in both of the devices due to the ferroelectric property of P(VDF-TrFE). However, the Fe-FET using DMF exhibits strong hysteresis. The threshold voltage shift is also an important factor in memory performance. As shown in Figure 4c, the 33.5 V and 29.8 V threshold values were shifted in Fe-FETs using DMF and MEK solvents, respectively. In addition, the Fe-FET using MEK solvent showed a low field-effect mobility of 0.09 cm^2^/Vs, while the Fe-FET using DMF solvent showed a high field-effect mobility of 0.12 cm^2^/Vs due to the low surface roughness of the P(VDF-TrFE) [27,28].

**Figure 5:**
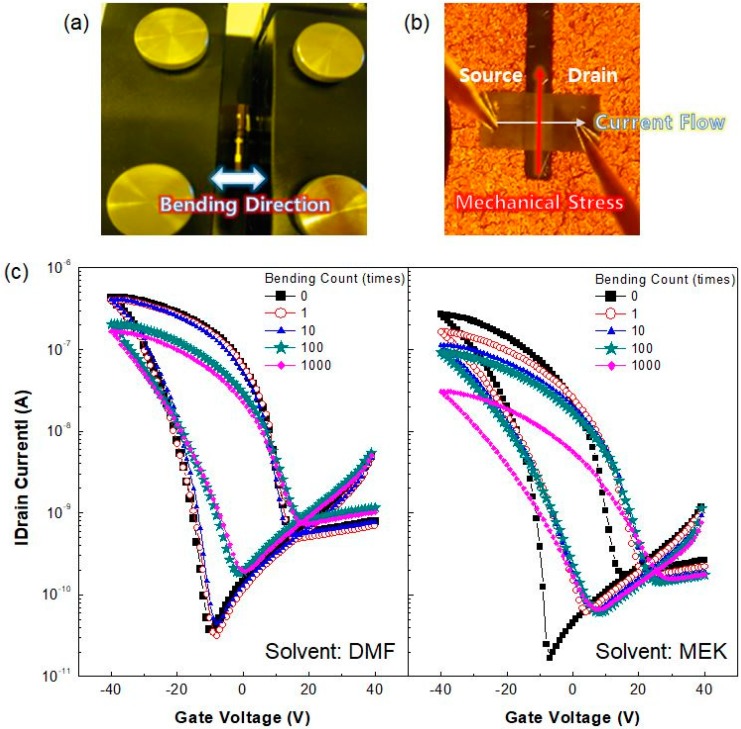
(**a**) Photograph of bending test of flexible Fe-FETs; (**b**) Microscopy image of Fe-FET being evaluated under bending; (**c**) Transfer characteristic curves of flexible Fe-FETs using DMF (left) and MEK (right) solvents before and after various bending cycles.

Figure 5a,b show a photograph of the bending test and a microscope image of an Fe-FET being evaluated while under bending, respectively. Tensile strain was applied to the Fe-FETs with a curvature radius of 10.2 mm perpendicular to the current flowing direction from the source to the drain electrodes. The variation of transfer characteristics in flexible Fe-FETs after repetitive bending is shown in Figure 5c. An Fe-FET from DMF solvent shows stable transfer characteristics up to 10 bending cycles, but degraded performance after over 1000 bending cycles. In contrast, FE-FETs using MEK solvents exhibited a significant decrease in electrical performance after only 1 bending cycle. In particular, Fe-FETs using MEK showed severe degradation in electrical performance with increasing bending cycles and were more susceptible to mechanical stress than those using DMF. 

**Figure 6:**
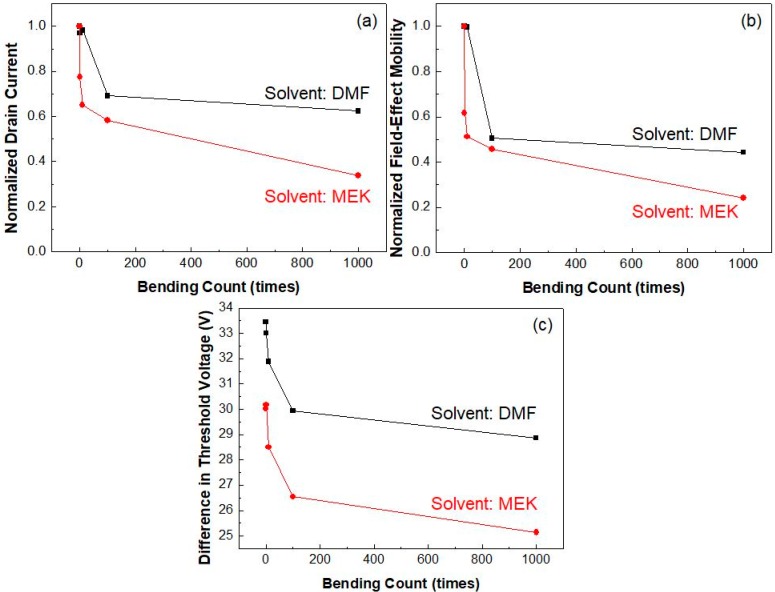
(**a**) Normalized drain current, (**b**) normalized field-effect mobility, and (**c**) difference in threshold voltage of flexible Fe-FETs using DMF (black) and MEK (red) solvents as a function of bending counts.

We compared the electrical and memory properties of Fe-FETs fabricated with DMF and MEK solvents after repetitive bending stress to understand the effect of solvents on the mechanical stability of these devices. Figure 6a–c show the normalized drain current, the normalized field-effect mobility, and the threshold voltage differences between flexible Fe-FETs from DMF and MEK solvents as a function of bending count. On-current and field-effect mobility gradually decreased with an increased bending count. In particular, Fe-FETs using MEK showed a significant degradation in on-current and field-effect mobility due to mechanical stress. After 1000 bending cycles, the on-current and field-effect mobility of MEK Fe-FETs decreased about 67% and 76%, respectively, compared to their initial values in Figure 6a,b. Fe-FETs using DMF showed 38% and 56% degradation in on-current and field-effect mobility, respectively, due to their low surface roughness. The threshold voltage, an important memory device parameter, when compared between the two types of Fe-FETs, showed similar decreases with increased bending count regardless of the solvent type, as shown in Figure 6c. This indicates that the hysteresis instability from mechanical stress is minimally affected by the type of solvent. These results reveal that mechanical stress predominately affects pentacene and P(VDF-TrFE) interface properties rather than ferroelectric properties, which are the origin of hysteresis. In summary, the insulator of Fe-FET using MEK has high surface roughness, thus deteriorating the charge transport property by forming physical and chemical defects after repeated mechanical stress.

## 4. Conclusions

We investigated the electrical properties and mechanical stability of flexible Fe-FETs for next-generation memory devices. In particular, the effect of solvent, which is one of the key components that determines the chemical and physical properties of thin-film, was studied using two solvents with different boiling points. The RMS roughness of P(VDF-TrFE) films from DMF and MEK solvents were 14.95 nm and 23.24 nm, respectively. Because of the surface roughness property, larger hysteresis, and higher field-effect mobility were obtained from Fe-FETs using DMF than from those using MEK, even though larger grain was observed in the MEK P(VDF-TrFE) devices. Furthermore, Fe-FETs using MEK solvent exhibited a significant degradation in electrical properties from bending stress. After 1000 bending cycles, the on-current and field-effect mobility of Fe-FETs using MEK decreased about 67% and 76%, respectively, compared to their initial values, while the same properties of Fe-FETs using DMF decreased about 38% and 56%, respectively. These results indicate that solvent selection is an important part of the fabrication of high-performance and mechanically stable flexible Fe-FETs.
